# Droplet size and surface hydrophobicity enhance bacterial plasmid transfer rates in microscopic surface wetness

**DOI:** 10.1038/s43705-022-00159-8

**Published:** 2022-08-16

**Authors:** Tomer Orevi, Søren J. Sørensen, Nadav Kashtan

**Affiliations:** 1 Department of Plant Pathology and Microbiology, Institute of Environmental Sciences, Robert H. Smith Faculty of Agriculture, Food, and Environment, Hebrew University, Rehovot, 76100 Israel; 2 Department of Biology, University of Copenhagen, DK 2100 Copenhagen, Denmark

**Keywords:** Microbial ecology, Soil microbiology

## Abstract

Conjugal plasmids constitute a major engine for horizontal gene transfer in bacteria, and are key drivers of the spread of antibiotic resistance, virulence, and metabolic functions. Bacteria in terrestrial habitats often inhabit surfaces that are not constantly water-saturated, where microscopic surface wetness (MSW), comprised of thin liquid films and microdroplets, permanently or intermittently occurs. How physical properties of microdroplets, and of the surfaces they reside on, affect plasmid transfer rates is not well understood. Here, building on microscopy-based microdroplet experiments, we examined the relation between droplet properties (size and spread) and plasmid transfer rates at single-cell and individual droplet resolution, using *Pseudomonas putida* as a model species. We show that transfer rates increase with droplet size, due to higher densities of cells on the surface in larger droplets, resulting from lower ratio between the area of the liquid-solid interface and droplet volumes. We further show that surface hydrophobicity promotes transfer rates via the same mechanism. Our results provide new insights into how physical properties of surfaces and MSW affect plasmid transfer rates, and more generally, microbial interactions mediated by cell-to-cell contact, with important implications for our understanding of the ecology and evolution of bacteria in unsaturated environments.

Conjugative plasmids confer countless important bacterial traits, including antibiotic resistance and pathogenicity, and are a major vehicle of horizontal gene transfer within microbial communities [1–5]. In terrestrial ecosystems, bacteria commonly inhabit surfaces that are not constantly saturated with water, which are often covered by thin liquid films and microdroplets, termed microscopic surface wetness (MSW) [6–8]. Under MSW conditions, cells are confined within droplets or films for prolonged periods, and thus cell-to-cell interactions mediated by physical contact, which is required for conjugation, are expected to be high. Conjugation also depends upon the cells’ physiological state [9–11], which is in turn affected by the unique physicochemical conditions of MSW [6]. A number of studies have elucidated the mechanisms that modulate plasmid transfer rates on surfaces and in porous media at the microscale [12–15], as well as the various ways in which hydration conditions and dynamics affect conjugation rates [10, 12, 16, 17]. Yet, how basic physical properties of individual microdroplets (e.g., their dimensions) and those of the surfaces upon which they reside, affect plasmid transfer rates, has not been studied in depth.

Here, we use a microscopy-based microdroplet experimental system that enables us to track plasmid transfers within individual microdroplets, after spraying droplets of media-suspended bacterial cells onto a glass surface (see Methods in SI). We use strains of *Pseudomonas putida* KT2440 [18] – a well-studied model bacterium for unsaturated environments – as plasmid donors and recipients. The donor strain carries a conjugal plasmid with a GFP reporter that is repressed in the donor cells, but expressed in trans-conjugant cells [4].

We first asked how droplet size affects the number of plasmid transfer events (T_e_) within individual droplets. We sprayed bacteria suspended in liquid M9 minimal media onto glass-bottom 12-well plates. Then, plates were kept at 28 °C and at relative humidity (RH) of ≈98% (SI). We captured microscopy images of the wells’ bottom surfaces at 0, 3, 6, 12, and 18 h post inoculation. Under these conditions, evaporation rate was slow and droplets did not dry out (Fig. 1A, Fig. S1), while cells within them grew very slowly (Table S1). The area of the liquid-solid interface of each sessile droplet (the contact area between the droplet and the glass surface, which we term ‘droplet area’) was used as a quantitative measure of droplet size. Droplet area spanned several orders of magnitude ranging from ~10^2^ µm^2^ to ~10^5^ µm^2^ (Fig. 1A, B).

**Fig. 1:**
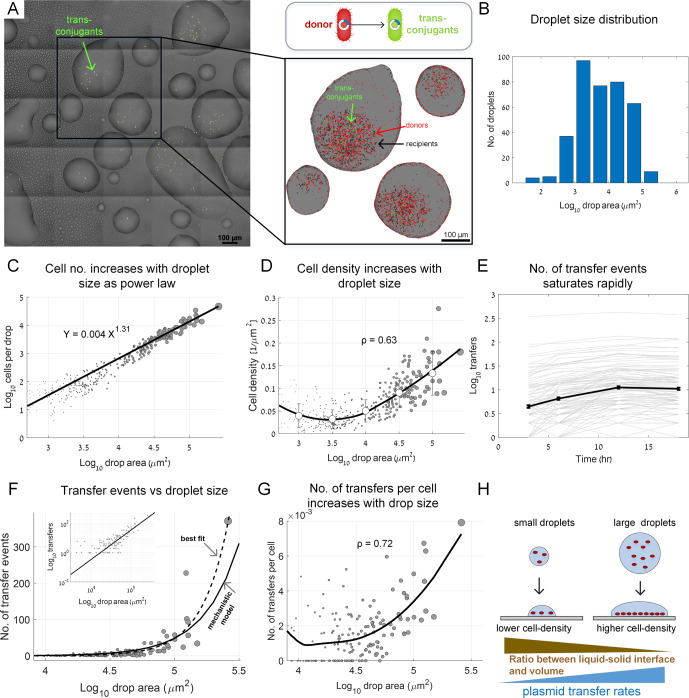
Droplet size effect on plasmid transfer rates. **A** A representative section of the glass surface covered by sprayed droplets at 6 h after spraying. Green cells are trans-conjugants. Inset: zooming into individual droplets. Donor, recipient, and trans-conjugant cells are shown in red, black, and green respectively. **B** Droplet area histogram (‘area’ refers to the area of the liquid <> solid interface, *A*). **C** The total number of cells (*N*) in individual droplets (including donors, recipients, and trans-conjugates at *t* = 6 h). Circle size reflects droplet area. Axis plotted in log-log scale (dataset includes 372 droplets). **D** Surface cell density in individual droplets. Line shows smoothed average (using LOESS), circles represent mean ± STD for binned data. ρ represents Spearman rank correlation coefficients (*P* < 10e-10). **E** Lines show cumulative number of transfer events over time in individual droplets at 3, 6, 12, and 18 h post inoculation (semi-log scale). Solid black line represents mean ± SE (SE smaller than symbols). **F** Number of transfer events as a function of droplet area: Circles are experimental data. Black line: mechanistic model *T*_*e*_ = 3.58·10^−7^·*A*^1.62^ (SI). Dashed line: best-fitted line based on data *T*_*e*_ = 1.08·10^−9^·*A*^2.12^ (fitted as a power law, SI). Inset: same data, but at log-log scale. **G** Transfer events per cell (*T*_*c*_) as a function of droplet area. Black line is smoothed data (LOESS). ρ represents Spearman rank correlation coefficients (*P* < 10e-10). **H** Conceptual representation of the underlying mechanisms explaining the increase in plasmid transfers as a function of droplet area. In (**F**) and (**G**), droplets are shown only for *A* > 10^3.9^ µm^2^ (smaller droplets show mostly zero transfers).

We focused our analyses on the liquid-solid interface, where we observed that the majority of cells settle and where most plasmid transfer events occurred. The number of cells (*N*) observed on the surface in each droplet increased as a power function of droplet area (*A*) with a slope >1 (*N* = 0.004·*A*^1.31^; Fig. 1C), reflecting a lower ratio of (*A*) to droplet volume (*V*) in larger droplets. Importantly, though initially slightly decreasing, surface cell density increased with droplet area for droplets > 3000 µm^2^ (Fig. 1D). Assuming equal cell densities in the sprayed droplets prior to surface deposition, in larger droplets a higher number of cells are deposited on a smaller area of the surface, leading to increased cell density (Fig. 1D). We hypothesized that the increased density in larger droplets would result in a higher number of transfers per cell (*T*_*c*_).

We first tracked the number of transfer events (*T*_*e*_) within individual droplets. Trans-conjugates were observed as early as a few hours post inoculation. At 6 h, there were already dozens of transfer events in the larger droplets (Fig. 1A, E), the majority occurring within 6–12 h, peaking at 12 h (Fig. 1E), corroborating other studies [10, 16, 17]. The data indicate that the accumulated number of plasmid transfer events per droplet behaves similarly at the various time points (reflected in a power function with similar slopes, Fig. S2). We decided to focus on the t = 6 h time point for further in-depth analysis.

Next, we sought to test whether *T*_*e*_ is indeed governed by cell density. Theoretically, in each droplet, the number of donor and recipient cells per unit area dictate the probability of donor- recipient physical contact (that is required for conjugation). As cells did not always remain in an exact location throughout the experiment, instead of counting donor-recipient pairs exhibiting physical contact, we employed a statistical approach utilizing data from hundreds of individual droplets for fitting a simple per-droplet, population-based mechanistic model [1, 19]:

1 $$T_e = k \cdot \left( {D_d \cdot D_r \cdot A} \right)$$

where *D*_*d*_ – donor density; *D*_*r*_ – recipient density; *A* – drop area; and *k* is a constant (see more details in SI). Indeed, analysis of a subset of droplets, wherein we counted all donor-recipient pairs within a 5 µm distance, showed that such a population-based approach can serve as a good approximation for the number of donor-recipient pairs that are located in close proximity (Fig. S3).

To develop a mechanistic model for *T*_*e*_ as a function of droplet area (*A*), we first approximated *D*_*d*_ and D_r_ based on the empirical power-law relation between the expected number of cells (*N*) and A

2 $$N = \beta 1A^{\alpha 1}$$

(Figure 1C), and took into account the overall fraction of donor and recipient cells (*p*,1-*p* respectively; here *p* ≈ 0.4; see also Fig. S4). Substituting Eq. (2) into Eq. (1) yields the following model for *T*_*e*_ (see details in SI):

3 $$T_e = k \cdot p\left( {1 - p} \right) \cdot \beta 1^2 \cdot A^{(2\;\alpha 1 - 1)}$$

We then fitted the above mechanistic model to the empirical *T*_*e*_ data in individual droplets by fitting the scale factor *k* (Fig. S5, SI). This yielded the power function *T*_*e*_ = 3.58·10^−7^·*A*^1.62^ (see SI), which agrees well with the experimental data (Fig. 1F; see also inset). A best-fitted power-law model, that was not based on our mechanistic model, showed a somewhat higher exponent: *T*_*e*_ = 1.08·10^−9^·*A*^2.12^ (Fig. 1F, SI), possibly indicating an additional positive effect of *A* on *T*_*e*_, or a higher ‘effective’ density within larger droplets (as can be seen in Fig. 1A inset and Fig. S1, possibly due to droplet growth by condensation). We moreover found that not only does *T*_*e*_ increase with *A*, but so does the mean number of transfers per cell (*T*_*c*_) (Fig. 1G) as expected by a similar mechanistic model for *T*_c_ (SI, Fig. S6). These results highlight the apparent underlying mechanism: a decrease in *A*/*V* leading to an increase in surface cell density with droplet size (Fig. 1H).

As the ratio *A*/*V* appears to be a key variable of the system, we next sought to test how surface hydrophobicity affects the system. We hypothesized that a more hydrophobic surface would lead to a higher *T*_*c*_ due to a lower *A*/*V* ratio that results in higher cell densities, due to the reduced spreading and more spherical droplet shapes with decreased wettability (illustrated in Fig. 2I). To test this hypothesis, we modified the hydrophobicity of our glass-bottom well plates to produce a more hydrophobic or a hydrophilic surface (contact angles of ≈90° and ≈42° respectively, see SI and Fig. S7). We then performed similar microdroplet experiments with the modified surfaces (due to technical reasons, we also changed additional settings of the original experiment, SI).

**Fig. 2:**
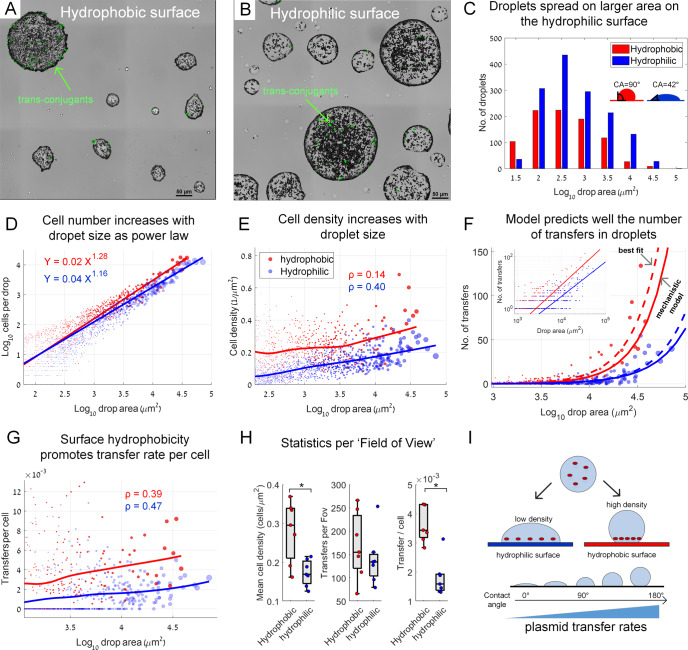
Surface hydrophobicity effect on plasmid transfer rates. **A** A representative section of a hydrophobic surface covered by sprayed droplets at 6 h after spraying. Green cells are trans-conjugants. **B** Same as in (**A**), but on a hydrophilic surface. **C** Droplet area histograms (area refers to area of the liquid-solid interface). Contact angles (*CA*) were measured as described in SI. **D** Cell numbers in individual droplets including donors, recipients, and trans-conjugates. Circle size reflects droplet area. Axis plotted on a log-log scale (dataset includes 2518 droplets). **E** Surface cell density in individual droplets. Line represents average smoothing (LOESS). ρ represents Spearman rank correlation coefficients (*P* = 0.0294 for the hydrophobic experiments, and *P* < 10e-10 for the hydrophilic one; ρ calculated for drops with *A* > 10^3^ µm^2^). **F** Number of transfer events (*T*_*e*_) as a function of droplet area: Circles represent experimental data. Solid lines: mechanistic model (see SI). Dashed line: best-fitted line based on data (fitted as a power function, SI). Inset: same data, but at log-log scale. **G** Transfer events per cell (*T*_*c*_) as a function of droplet area. Solid lines represent average smoothing (LOESS). ρ represents Spearman rank correlation coefficients (*P* = 3.44e-10 for the hydrophobic experiment, and *P* < 10e-10 for the hydrophilic one; ρ calculated for drops with *A* > 10^3^ µm^2^). **H** Statistics per entire surface sections (each of 1.6 mm × 1.6 mm). Boxplot represents median and 25%, 75% percentiles. Statistical comparisons were based on two-sample *t*-test yielding *P* = 0.0058, *P* = 0.41, and *P* = 1.34e-04 for the three comparisons respectively. * indicates statistical significance at 0.01 significance level. **I** Conceptual representation of the underlying mechanisms explaining the increase in plasmid transfer rates as a function of surface hydrophobicity. In (**F**) and (**G**), droplets are shown only for *A* > 10^3^ µm^2^ (smaller droplets show mostly zero transfers).

The average area of the sprayed droplets on the hydrophilic surface was indeed about twice that of the hydrophobic one (Fig. 2A, B) as reflected in the droplet area distributions (Fig. 2C, Fig. S8). The number of cells per droplet reflected that as well: There were more cells per given droplet area on the hydrophobic surface (Fig. 2D). Once again, cell density increased with droplet area, with higher densities on the hydrophobic surface (Fig. 2E).

*T*_*e*_ increased with *A* for both surface types, corroborating our previous experiment (Fig. 2F). We found that the mechanistic model, with a fitted *k* (Fig. S9) and incorporating the fitted power function of *N* as a function of *A* (Fig. 2D), agreed well with the observed *T*_*e*_ data (Fig. 2F, SI). Importantly, the fitted value of *k* for both surface types was very similar (≈0.035), supporting the validity of our model and hypothesis that the relationship between *N* and *A* dictates *T*_*e*_. Again, a best-fitted model based on data showed only a slightly higher increase in *T*_*e*_ as a function of *A* (Fig. 2F, SI). As before, *T*_*c*_ increased with *A* and was higher for the hydrophobic surface, as anticipated (Fig. 2G). The increase in *T*_*c*_ on the hydrophobic surface was weaker for small droplets, likely due to higher evaporation of small droplets under these conditions (see also Fig. S1). The described general trends were validated with a cumulative analysis of the entire surface sections that were scanned (Fig. 2H). In summary, in addition to droplet size, surface hydrophobicity promotes *T*_*e*_ through increasing surface cell densities (Fig. 2I).

The demonstrated relation between droplet physics and plasmid transfer rates has implications for diverse microbial habitats, where MSW is common, including the built environment, soil, root and leaf surfaces [8], and animal and human skin. In many of these habitats, cells frequently arrive on surfaces within microdroplets. For example, in the built environment, microdroplets expelled from the human respiratory tract settle on inanimate surfaces. In the phyllosphere, bacteria often arrive on leaf surfaces via rain or agricultural spraying (e.g., in biocontrol application). In these cases, the physics underlying the increase of cell densities with droplet size, or with surface hydrophobicity, as demonstrated in our *in vitro* system, is expected to be valid. Nonetheless, other physical processes may lead to additional effects such as flow and pattern formation (in particular in macroscopic drops [17]) that might depend also on the chemical composition of solutions and physical properties of the environment (e.g., surface micro-topography). Clearly, bacterial colonization of unsaturated surfaces in nature may involve arrival of cells via other (than droplet deposition) means. Yet, higher bacterial densities in larger droplets are likewise expected not only in the case of deposition of droplets, but also due to other mechanisms, including favorable growth conditions and higher survival in larger droplets [7], and synergistic inter-species interactions due to higher diversity in larger droplets [20]. This suggests that in real-world conditions, this link between cell densities and droplet size might even be stronger, thus leading to more pronounced increase in *T*_*e*_ as a function of *A*.

To conclude, our results provide new insights into the ways physical properties of surfaces and MSW affect conjugal plasmid transfer rates. We show that transfer rates in MSW are governed by cell density, which in turn, is dictated by droplet area and surface hydrophobicity. Our results further demonstrate how droplet physics and microscale wetting properties affect plasmid transfer rates, and more broadly, affect interactions that require cell-to-cell physical contact, with important implications for the ecology and evolution of bacteria in unsaturated environments.

## Supplementary information

Supporting Information

## Data Availability

Data is available at figshare. Dataset. 10.6084/m9.figshare.19826407.v1. Microscopy images are available upon request.

## References

[1] Lopatkin AJ, Sysoeva TA, You L (2016). Dissecting the effects of antibiotics on horizontal gene transfer: Analysis suggests a critical role of selection dynamics. Bioessays.

[2] Norman A, Hansen LH, Sørensen SJ (2009). Conjugative plasmids: Vessels of the communal gene pool. Philosophical Transac Royal Soc B: Biological S.

[3] Harrison E, Brockhurst MA (2012). Plasmid-mediated horizontal gene transfer is a coevolutionary process. Trends Microbiol.

[4] Klümper U, Riber L, Dechesne A, Sannazzarro A, Hansen LH, Sørensen SJ (2015). Broad host range plasmids can invade an unexpectedly diverse fraction of a soil bacterial community. ISMEJ.

[5] Heuer H, Smalla K (2012). Plasmids foster diversification and adaptation of bacterial populations in soil. FEMS Microbiol Rev.

[6] Orevi T, Kashtan N (2021). Life in a Droplet: Microbial Ecology in Microscopic Surface Wetness. Front Microbiol.

[7] Grinberg M, Orevi T, Steinberg S, Kashtan N (2019). Bacterial survival in microscopic surface wetness. eLife.

[8] Burkhardt J, Hunsche M (2013). “Breath figures” on leaf surfaces—formation and effects of microscopic leaf wetness. Front Plant Sci.

[9] Seoane J, Yankelevich T, Dechesne A, Merkey B, Sternberg C, Smets BF (2011). An individual-based approach to explain plasmid invasion in bacterial populations. FEMS Microbiol Ecol.

[10] Normander B, Christensen BB, Molin S, Kroer N (1998). Effect of bacterial distribution and activity on conjugal gene transfer on the phylloplane of the bush bean (Phaseolus vulgaris). Appl Environmental Microbiol.

[11] Fox RE, Zhong X, Krone SM, Top EM (2008). Spatial structure and nutrients promote invasion of IncP-1 plasmids in bacterial populations. ISMEJ.

[12] Tecon R, Ebrahimi A, Kleyer H, Levi SE, Or D (2018). Cell-to-cell bacterial interactions promoted by drier conditions on soil surfaces. Proc Natl Acad Sci.

[13] Lagido C, Wilson IJ, Glover LA, Prosser JI (2003). A model for bacterial conjugal gene transfer on solid surfaces. FEMS Microbiol Ecol.

[14] Merkey BV, Lardon LA, Seoane JM, Kreft JU, Smets BF (2011). Growth dependence of conjugation explains limited plasmid invasion in biofilms: an individual‐based modelling study. Environmental Microbiol.

[15] Gu H, Kolewe KW, Ren D (2017). Conjugation in Escherichia coli biofilms on poly (dimethylsiloxane) surfaces with microtopographic patterns. Langmuir..

[16] Björklöf K, Nurmiaho‐Lassila EL, Klinger N, Haahtela K, Romantschuk M (2000). Colonization strategies and conjugal gene transfer of inoculated Pseudomonas syringae on the leaf surface. J Appl Microbiol.

[17] Ruan C, Ramoneda J, Chen G, Johnson DR, Wang G (2021). Evaporation-induced hydrodynamics promote conjugation-mediated plasmid transfer in microbial populations. ISME Commun.

[18] Nelson K, Weinel C, Paulsen I, Dodson R, Hilbert H, Martins dos Santos V (2002). Complete genome sequence and comparative analysis of the metabolically versatile Pseudomonas putida KT2440. Environmental Microbiol.

[19] Levin BR, Stewart FM, Rice VA (1979). The kinetics of conjugative plasmid transmission: fit of a simple mass action model. Plasmid..

[20] Liu W, Russel J, Burmølle M, Sørensen SJ, Madsen JS (2018). Micro-scale intermixing: a requisite for stable and synergistic co-establishment in a four-species biofilm. ISME J.

