# Supplementary material for: Droplet size and surface hydrophobicity enhance bacterial plasmid transfer rates in microscopic surface wetness
Source: ISME Commun. 2022 Aug 16;2:72. doi: 10.1038/s43705-022-00159-8 (PMC9723546; doi:10.1038/s43705-022-00159-8)
Supplement: Supplementary file 1 — Supporting Information [file 43705_2022_159_MOESM1_ESM.pdf]

Supporting Information

Droplet size and surface hydrophobicity enhance bacterial plasmid transfer rates in microscopic surface wetness

Orevi et al.

SI includes:

1. Supporting Materials and Methods

2. Supporting Figures

3. Supporting Tables

1. Supporting Materials and Methods

Bacterial strains and growth conditions

Pseudomonas putida KT2440 cells were used as donor and recipient pair. Donor cells were constructed by Sørensen & Smets as described elsewhere [1]. Briefly, donor cells are chromosomally tagged with constitutively expressed mCherry and lacIq production (and kanamycin resistance gene). Additionally, donor cells carry a broad host pKJK5 plasmid marked with gfpmut3b gene, which is expressed by a LacIq repressible promoter Plac (plasmid includes kanamycin and tetracycline resistance genes). *P. putida* KT2440 cells were routinely cultured in M9 medium (M9 Minimal Salts Base 5x, Formedium, UK) supplemented with 20 mM Glucose and 50 µg/mL Km + 10 µg/mL tetracycline (for the donor strain), under agitation set at 220 rpm, at 28°C.

Experiment 1: Plasmid transfer in droplets sprayed on untreated glass substrate

Donor and recipient strain were cultured (separately) in 50 mL Falcon tubes continuing 25 mL of medium and appropriate antibiotic. Overnight cultures were washed twice (centrifuge at 6,000 rcf for 5 min) in M9 medium, and the pellets were re-suspended in 2 mL medium in order to reach high-density cultures (OD_{600} > 5). Next, the OD of the donor and recipient strains were adjusted to OD_{600} = 4, and the strains were mixed with a 1:3 (donor:recipient) ratio in a 5 mL tube that contained 1 µM of Alexa dye (Alexa Fluor 647, Invitrogen) that was used to fluorescently stain the sprayed droplets. The solution was loaded into 5 mL refillable spray bottles (purchased at a local cosmetics store), and a portion of the load was sprayed on a 12-well glass-bottom plate (P12-1.5H-N, Cellvis) in the following manner: a 12-well plate was placed (without the plastic lid cover) in a plastic bag, and then the solution was delivered by pressing the spray nozzle 4 times from a distance of about 15 cm above the plate. Tap water was added to the empty spaces between the wells of the plate, plates were covered with the plastic lid, and the plate's perimeter was sealed with a stretchable sealing tape to maintain a humid environment (>98% RH). The plate was incubated in the dark at 28ºC throughout the duration of the experiment (18 hrs).

Experiment 2: Plasmid transfer in droplets sprayed on hydrophobic and hydrophilic modified glass substrate

Experiment 2 was conducted similarly to Experiment I with the following modifications: (a) The donor and recipient mix solution was adjusted to $OD_{600} = 3$ at a 1:1 ratio (b) Spray was applied to each well separately through a cylinder made out of 50 mL Falcon tube from which the conical end was chopped 1.5 cm above the base of the tube (one squeeze of the spray nozzle per well) (c) Hydrophobic and hydrophilic modified glass well plates were used.

Glass modification

Procedure for hydrophobic and hydrophilic glass modification was adopted from [2] with a few adjustments: 12-well glass-bottom plates (P12-1.5H-N, Cellvis) were filled with 2% RBS35 solution (RBS™ 35 solution, Sigma) and sonicated for 5 min in an ultrasonic bath (model ACP-200H, MRC) followed by thorough rinsing with tap water, demineralized water, methanol, tap water, and finally demineralized water again to obtain a hydrophilic surface. The plates were dried in an oven for 2 hrs at 70ºC, and then either stored before use or further modified by applying a hydrophobic coating. To obtain a hydrophobic surface, 20 µL of 2% (v/v) dichlorodimethylsilane (CAS 75-78-5, Sigma) in trichloroethylene (CAS No 79-01-6, Sigma) were applied to the center of each well (avoiding contact between the siliconizing solution and the boundaries of the well, which would result in melting of the well plate coating) and left to dry for 1 hr in a chemical hood. The plates were then dried in an oven for 2 hrs at 70ºC, rinsed with tap water, demineralized water, and dried in the hood for 2 hrs before use.

Microscopy

On the indicated time points (see Main Text), 12-well plates were mounted on a stage top chamber (H301-K-FRAME, Okolab) set at 28°C. Microscopic inspection and image acquisition were performed using an Eclipse Ti-E inverted microscope (Nikon) equipped with Plan Apo 40x/095 N.A. air objective and the Perfect Focus System for maintenance of focus. An LED light source (SOLA SE II, Lumencor) was used for fluorescence excitation. GFP fluorescence was excited with a 470/40 filter, and emission was collected with a T495lpxr dichroic mirror and a 525/50 filter. mCherry fluorescence was excited with a 545/25 filter, and emission was collected with a T565lpxr dichroic mirror and a 605/70 filter. Alexa 647 fluorescence was excited with a 620/60 filter, and emission was collected with a T660lpxr dichroic mirror and a 700/75 filter. Filters and dichroic mirror were purchased from Chroma, USA. A motorized encoded scanning stage (Märzhäuser Wetzlar, DE) was used to collect multiple positions of the well bottom surface. In each well, two random positions were chosen and imaged by scanning 5 × 5 adjacent fields of view (with a 5% overlap, 1.6 × 1.6 mm per scan). Images were acquired with an SCMOS camera (ZYLA 19 4.2PLUS, Andor, Oxford Instruments, UK). NIS Elements 5.02 software was used for acquisition.

Image processing

Image processing and analyses were performed to quantify the area of the liquid-surface interface (droplet area), total number of cells per droplet, number of donor cells per droplet, and plasmid transfer events per droplet. NIS Elements 5.02 software was used for image processing. Droplet masks were generated by intensity threshold segmentation of the Alexa 647 channel. Binary masks were converted to region of interest (ROI) elements delineating droplets' boundaries. Bright field channel was used to identify the total cell (donor, recipient, and transconjugate) entities within single droplets (i.e., identified ROIs). Rolling ball background correction (0.49 µm) was applied on the entire image, and the 'spot detection tool' was applied to enumerate cell number. mCherry channel was used to identify the donor cell entities within single droplets. Rolling ball background correction (0.49 µm) was applied on the entire image, and 'spot detection tool' was applied to enumerate donor cell number. GFP channel was used to identify trans-conjugant cells within single droplets. Rolling ball background correction (0.49 µm) was applied on the entire image, and intensity threshold was applied to identify single GFP expressing cells. The 'dilate' tool was operated on the resulting binary mask in order to cluster adjacent GFP expressing cells into a single object counted as a single 'plasmid transfer event' (i.e., we assumed that the plasmid was acquired prior to cell division). See also Fig. S10.

Data and statistical analysis

Data and statistical analysis were performed with MATLAB version 2021b.

Experiment 1 (untreated glass surface): Dataset consisted of 12 surface sections of 1.6 mm × 1.6 mm (FoVs) from 6 different wells, with a total of 372 droplets. Power function coefficients were estimated using 'fitnlm' function to a simple power function of the form: $Y=bX^a$. Data smoothing was done using 'smooth' (X,Y,r,'loess') with r = 0.8 in Fig. 1D and r = 0.5 in Fig 1G. Spearman rank correlation coefficients in Fig. 1D: rho = 0.63 P < 10e-10; and in Fig. 1G : rho = 0.72 P < 10e-10.

Experiment 2 (hydrophilic vs. hydrophobic treated glass surfaces):

Hydrophobic surface: Dataset consisted of 7 surface sections of 1.6 mm × 1.6 mm (FoVs) from 4 different wells, with a total of 1,087 droplets (1,069 with at least one cell). Hydrophilic surface: Dataset consisted of 7 surface sections of 1.6 mm × 1.6 mm (FoVs) from 4 different wells, with a total of 2,125 droplets (1,449 with at least one cell). Data smoothing lines were done using 'smooth' (X,Y,r,'loess') with r = 0.5 Fig. 2E r = 0.3 Fig 2G. Spearman rank correlation coefficients in Fig. 2E : hydrophobic – rho = 0.14 P < 0.0294; hydrophilic – rho = 0.40 P < 10e-10 (for drop area > 10^3) and in Fig.2G: hydrophobic – rho= 0.385 P = 3.4412e-10; hydrophilic – rho = 0.466 P < 1.69e-29 (for drop area > 10^3). Statistical tests in Fig. 2H based on a Welch two-sample t-test yielded the following results at confidence level of 0.01: (1) h = 1; P = 0.0058; tstat: 3.3470; df: 12; sd: 0.0601; (2) h = 0; P = 0.4103; tstat: 0.8532; df: 12; sd: 64.2169 (3) h = 1; P = 1.3471e-04; tstat: 5.5071; df: 12; sd: 6.3414e-04

Mechanistic model

Density-based mechanistic model for the number of transfers per droplet (T_e):

A naïve mechanistic model assumes that the number of plasmid transfer events is a multiplication of the densities of donor and acceptor cells in each droplet and the droplet area, and some factor k.

$$(1)\ T_e = k\ (D_d \cdot D_r \cdot A)$$

Where:

D_d is the donor cell density (units: $1/\mu m^2$)

Dr is the recipient cell density (units: $1/\mu m^2$)

A is droplet area (units: μm^2)

k is a constant (units: $1/\mu m^2$)

To estimate k, we used a simple regression model Y ~ kX where Y is the number of transfer events per droplet (T_e) and X equals $D_d \cdot D_r \cdot A$.

Assuming that p is the fraction of donors of all cells, and the recipients' fraction is (1-p), and that $D=N/A$, we get:

$$(2)\ T_e = k\ (D_d \cdot D_r \cdot A) = k\ [(p \cdot N/A) \cdot (1-p)(N/A) \cdot A];$$

where N is the total number of cells (donor + recipient) in the droplet.

N is estimated as a power function of the form:

$$(3)\ N = \beta 1 A^{\alpha 1}$$

Overall T_e model:

$$T_e = k\ (D_d \cdot D_r \cdot A) = k\ [p\ N/A \cdot (1-p)\ N/A \cdot A) = k[(p\ (\beta 1 A^{\alpha 1})/A) \cdot ((1-p)\ (\beta 1 A^{\alpha 1})/A) \cdot A] =$$
$$T_e = k \cdot p(1-p) \cdot \beta 1^2 \cdot A^{(2\alpha 1-1)}$$

Thus the overall model of T_e is:

$$(4)\ T_e = k \cdot p(1-p) \cdot \beta 1^2 \cdot A^{(2\alpha 1-1)}$$

Density-based mechanistic model for the number of transfers per cell (T_c; as an alternative to model T_e):

Although we chose to model T_e, we also present here a model for Tc.

$$(5)\ T_c = k\ (D_d \cdot D_r \cdot A)/N = (k/N) \cdot (p \cdot N/A) \cdot (1-p)(N/A) \cdot A = k \cdot p \cdot (1-p)(N/A) = k \cdot p \cdot (1-p)(\beta 1 A^{\alpha 1-1})$$

Experiment 1 (untreated glass)

We first estimated *k* (see (1) below and Fig. S5) based on our data, and then replaced *N* based on our empirical model of cell number as a function of droplet area (see (2) and Fig. 1C). For comparison, we also computed a best fit model (this model is based on fitting to the data, not a mechanistic model) of T_e as a function of *A* (see (3)). Power function coefficients were estimated using 'fitnlm' function to a simple power function of the form: $Y = \beta X^{\alpha}$.

Overall T_e model development:

$T_e = k\,(D_d \cdot D_r \cdot A) = k[p(1\text{-}p)] \cdot \beta 1^2 \cdot A^{(2\,\alpha 1-1)} = 0.136 \cdot [0.24 \cdot 0.76] \cdot 0.0038^2 \cdot A^{(2.62-1)} = 3.58 \cdot 10^{-7}\,A^{1.62}$

(1) *Estimating k:*

Only droplets wherein the average bin value crossed the threshold to show a positive number of transfers where used to calculate the slope (see Fig. S5).

Experiment 1: fitting k
$k = 0.13619 \pm 0.0044114$ (SE)<br>No. of observations: 126<br>R-Squared: 0.869<br>p-value = 2.49e-60

(2) Cell numbers – to - drop-area model:

Drops without any cells were removed from the analysis.

Experiment 1: fitting α and β
$\beta = 0.003844 \pm 0.0010917$<br>$\alpha = 1.3093 \pm 0.02431$<br>No. of observations: 355<br>R-Squared: 0.896<br>p-value = 3.12e-188

(3) Best fitted power law model based only on data:

Experiment 1: fitting b1 and b2
b1= 1.0876e-09 ± 2.4986e-11<br>b2= 2.1293 ± 3.3129e-19<br>No. of observations: 372<br>R-Squared: 0.829<br>p-value = 7.55e-148

Overall T_c model development (as an alternative to model T_e):

Although we chose to model T_e, we also present here a model for Tc.

$T_c = k\ (D_d \cdot D_r \cdot A)/N = (k/N) \cdot [(p \cdot N/A) \cdot (1-p)(N/A) \cdot A] = k \cdot p \cdot (1-p)(N/A) = k \cdot p \cdot (1-p)(\beta 1 A^{\alpha 1-1})$

$Tc = 0.136 \cdot 0.24 \cdot 0.76 \cdot 0.0038 \cdot A^{(1.31-1)} = 9.42 \cdot 10^{-5} A^{0.31}$

Best fitted power law model for T_C based only on data:

Experiment 1: fitting b1 and b2 (*Tc* model)
b1= 4.7147e-06 ± 8.4098e-06<br>b2= 0.56282 ± 0.16534<br>Number of observations: 370<br>R-Squared: 0.0409<br>p-value = 1.1e-08

Experiment 2 (modified glass)

Overall T_e model from eq. (3) is

$T_e = k \cdot p(1-p) \cdot \beta 1^2 \cdot A^{(2\alpha 1-1)}$

Treatment 1 - Hydrophobic:

$T_e = k \cdot p(1-p) \cdot \beta 1^2 \cdot A^{(2\alpha 1-1)} = 0.035 \cdot 0.5 \cdot 0.5 \cdot 0.023^2 \cdot A^{(2.56-1)} = 4.62 \cdot 10^{-6} A^{1.56}$

Treatment 2 - Hydrophobic:

$T_e = k \cdot p(1-p) \cdot \beta 1^2 \cdot A^{(2\alpha 1-1)} = 0.036 \cdot 0.5 \cdot 0.5 \cdot 0.040^2 \cdot A^{(2.32-1)} = 1.44 \cdot 10^{-5} A^{1.32}$

(1) *Estimating k:*

Only droplets wherein the average bin value crossed the threshold to show a positive number of transfers where used to calculate the slope (see Fig. S5).

Treatment 1 - Hydrophobic	*Treatment 2 - Hydrophilic*
k = 0.035182 ± 0.0025019<br>Number of observations: 117<br>R-Squared: 0.557<br>p-value = 8.03e-27	k = 0.036097 ± 0.0013806<br>Number of observations: 161<br>R-Squared: 0.738<br>p-value = 1.21e-59

(2) Cell numbers – to - drop-area model:

Treatment 1 - Hydrophobic	*Treatment 2 - Hydrophilic*
β = 0.022876 ± 0.0044684<br>α = 1.2815 ± 0.019358<br>Number of observations: 812<br>R-Squared: 0.896<br>p-value = 0	β = 0.04042 ± 0.0043457<br>α = 1.1632 ± 0.010485<br>Number of observations: 1,430<br>R-Squared: 0.909<br>p-value = 0

(3) Best fitted power law model based on data:

Treatment 1 - Hydrophobic	*Treatment 2 - Hydrophilic:*
b1 = 8.6092e-06 ± 3.1009e-06<br>b2 = 1.5385 ± 0.035257<br>Number of observations: 1,063<br>R-Squared: 0.795<br>p-value = 0	b1 = 6.7335e-06 ± 1.8278e-06<br>b2 = 1.4173 ± 0.025878<br>Number of observations: 1,515<br>R-Squared: 0.692<br>p-value = 0

Experiment 1. untreated glass

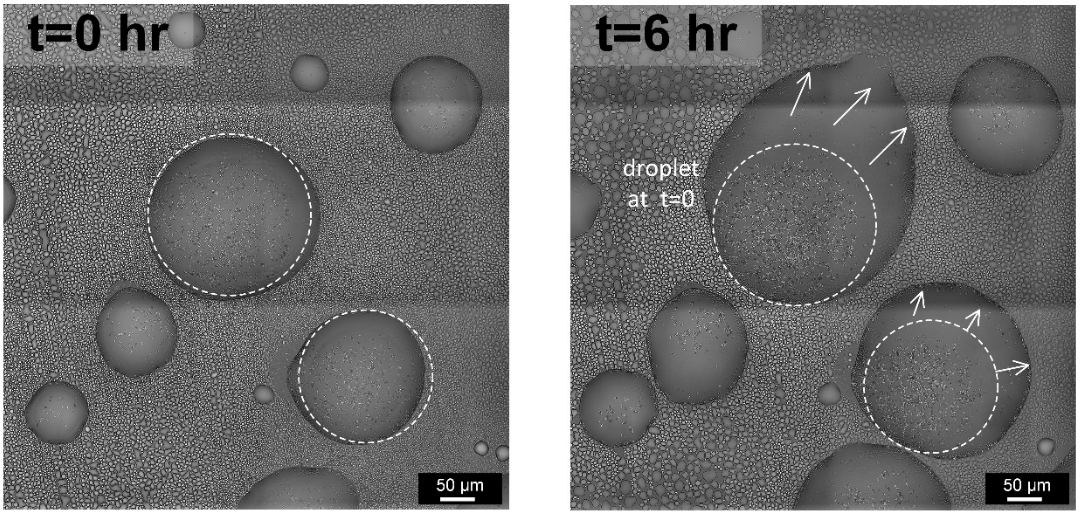

Experiment 2. hydrophilic

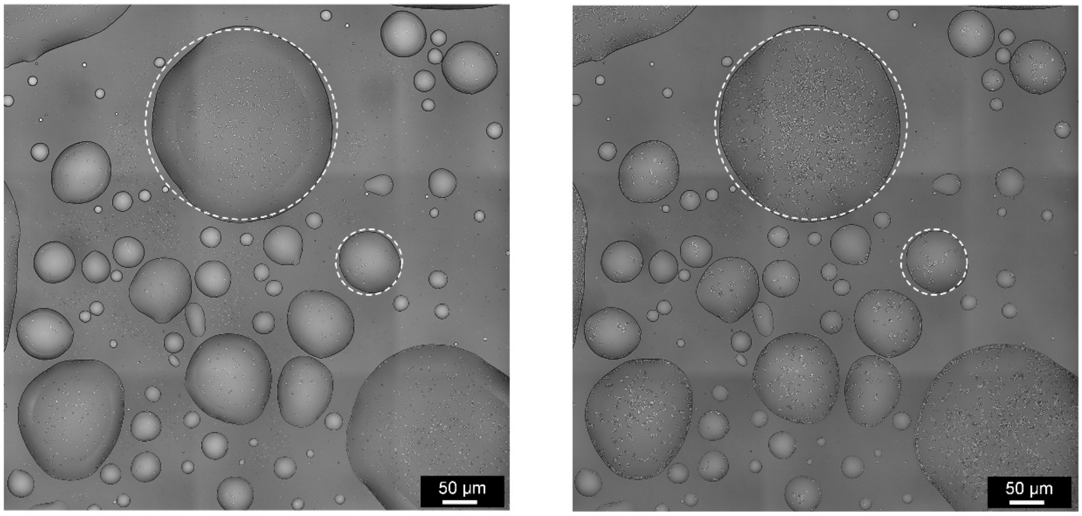

Experiment 2. hydrophobic

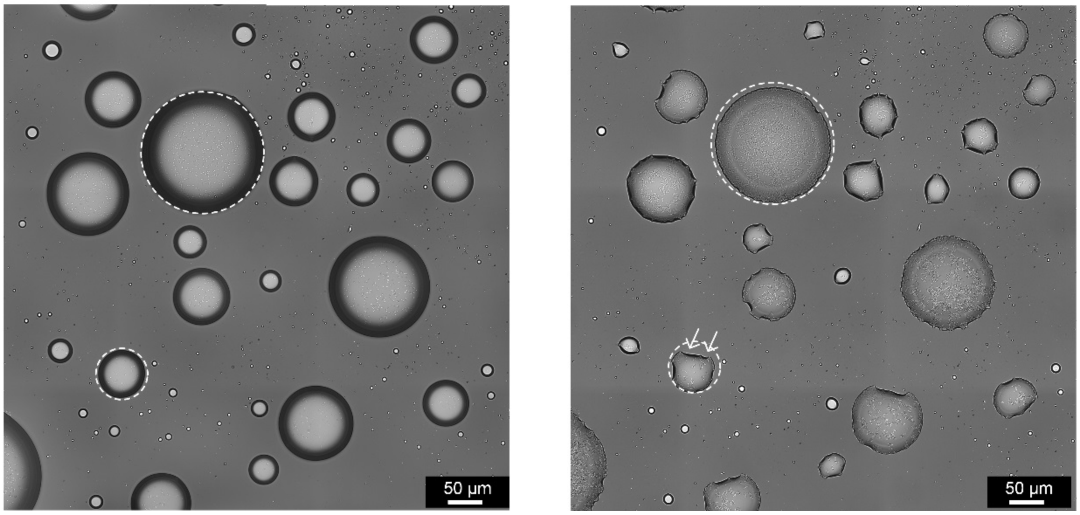

Fig. S1. Droplet evolution between t = 0 hr (spray deposition) and t = 6 hrs post deposition. Left column shows representative images captured immediately after spray application on untreated glass (Experiment 1), hydrophilic, and hydrophobic treated glass (Experiment 2). Right column shows the same sections of the surface 6hrs after spray delivery. Note that in Experiment 1 (left panels), droplets become larger due to condensation or spread, while in Experiment 2 (middle and right panels) droplet size remained more stable on the hydrophilic surface, and shrank a bit due to evaporation on the hydrophobic surface. Note that evaporation was somewhat higher in smaller droplets. All images show an 800 X 800μm section.

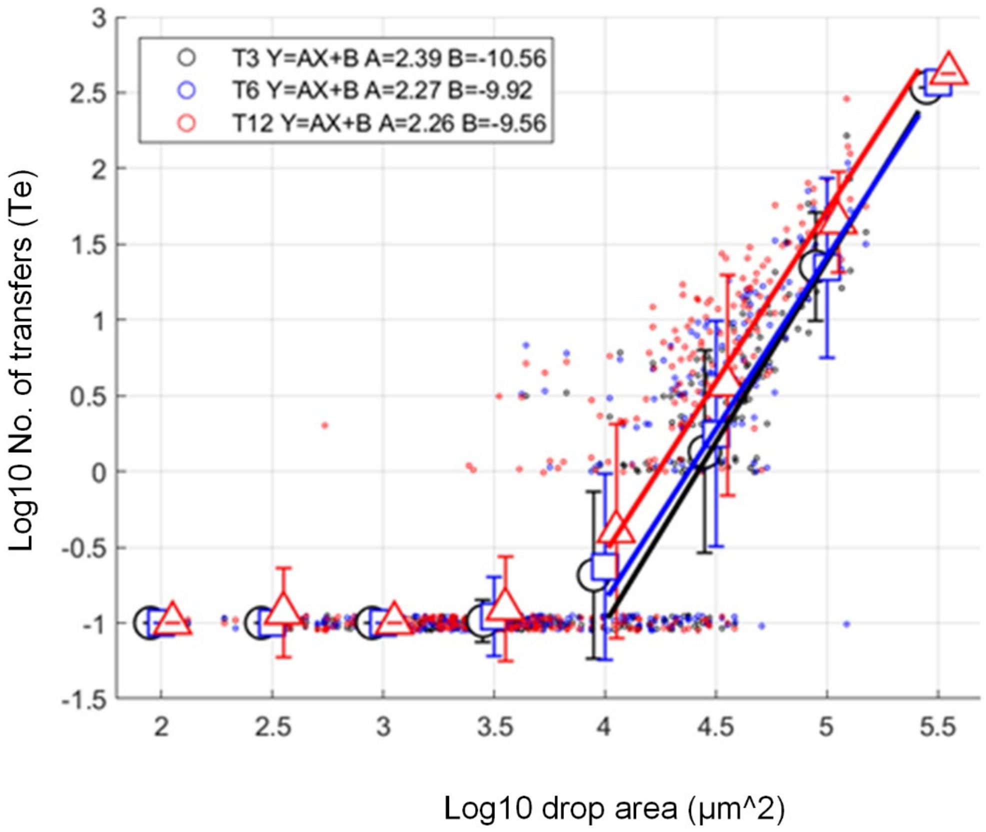

Fig. S2. Plasmid transfer event (T_e) at the three time points: 3hrs (black circle), 6hrs (blue square), 12hrs (red triangle) follow the same pattern. Similar exponent values of (≈2.3) of the power function were observed between time points. These data indicate that the plasmid transfer events show similar dynamic patterns along these time points and across the entire range of droplet areas.

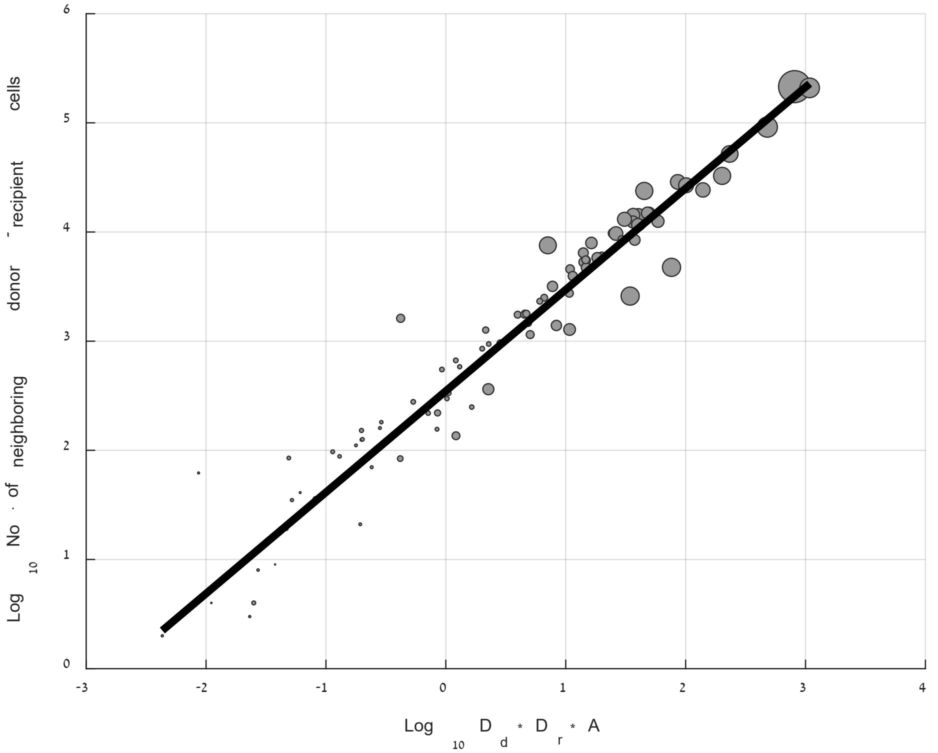

Fig. S3. The number of neighboring donor and recipient cells within droplet vs. mechanistic density per-droplet population-based model. Data is shown for a subset of 106 droplets from Experiment 1 (untreated glass). In each droplet, the entity of each cell was determined (donor or recipient) as well as the exact x,y coordinates on the surface. The number of cells within a neighborhood of 5µm was computed for each donor cell (no. of neighbors). The X-axis depicts the naïve 'population-based' mechanistic model that we evaluated in the main text. Drops without any cells were removed from the analysis. Power function coefficients were estimated using 'fitnlm' function to a simple power function of the form: $Y = \beta' X^{\alpha'}$. This yielded the following fit: $Y = 349 \cdot X^{0.93}$ with an R^2 of 0.97. This analysis shows that the density-based model provides a good approximation for the number of donor-recipient pairs in close proximity.

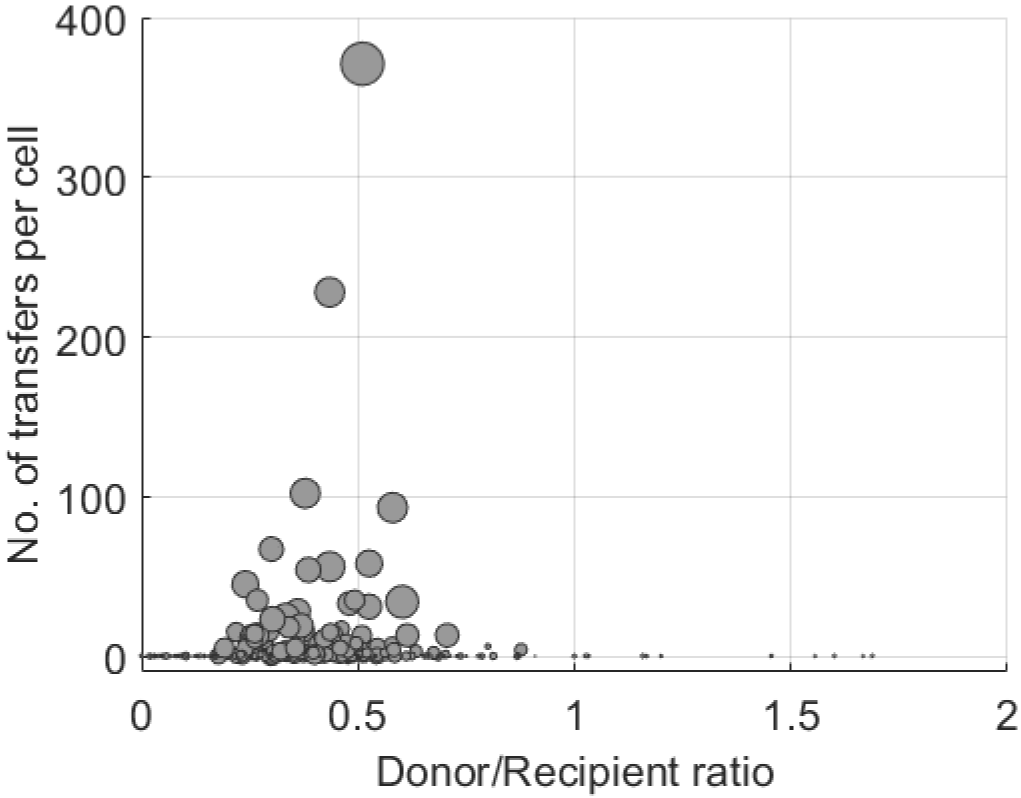

Fig. S4. No. of transfer events per droplet as a function of Donor:Recipient ratio. Circles are experimental data. Circle size represents droplet area. The distribution of ratio values for these droplets is 0.406±0.349 (mean±SD). The ratio in most middle- to large-size droplets is around the mean, while it significantly deviates only in very small droplets.

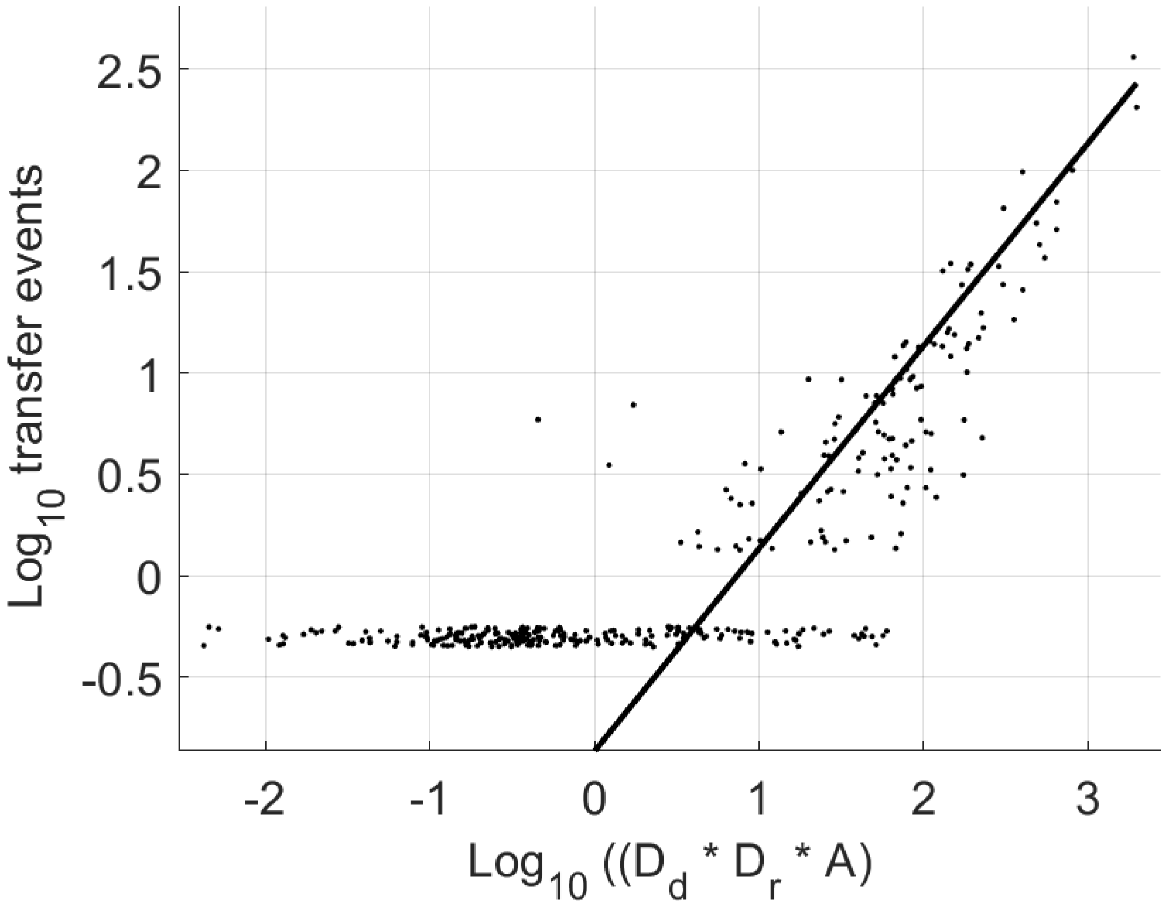

Fig. S5. Mechanistic Density-based model of Transfer events in Experiment 1 (t = 6hrs). Axes are in log-log scales. Straight line describes fitted model based on 'fitnlm' Matlab function to $Y = kX$. $k = 0.136 \pm 0.004$ (SE). Black dots represent experimental results.

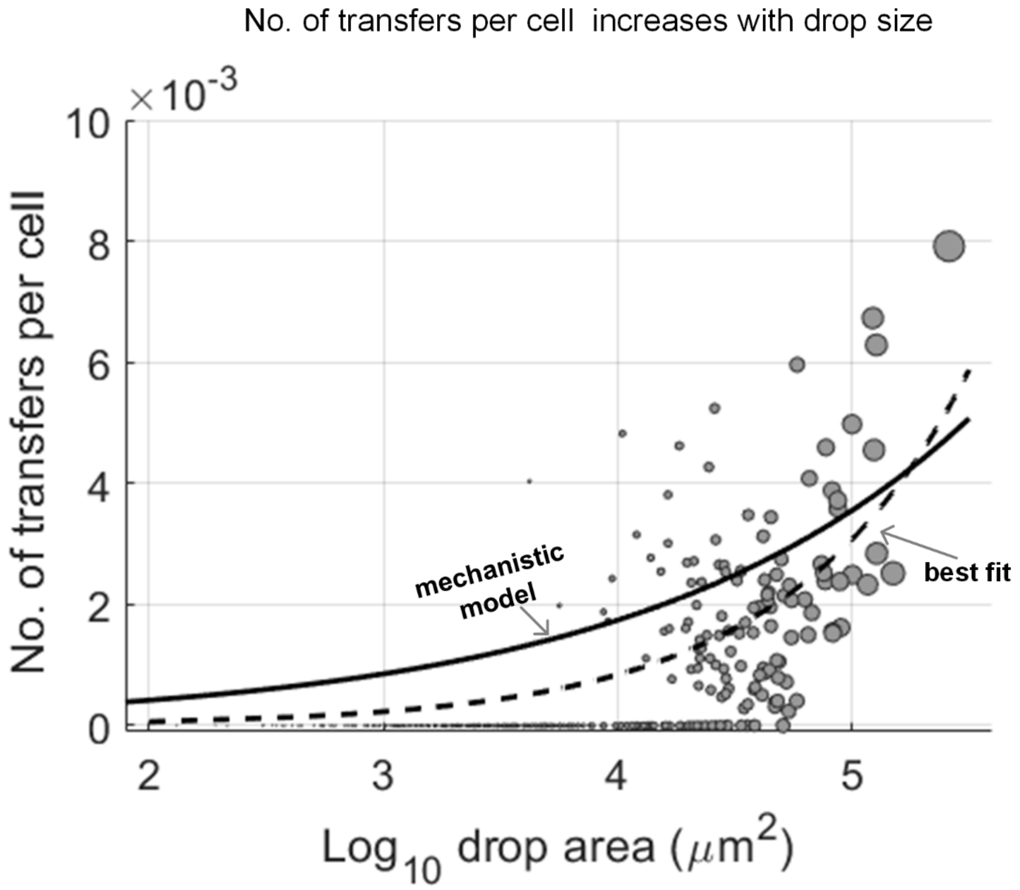

Fig. S6. Mechanistic density-based model of number of transfer rate per cell (T_c) in Experiment 1 (t = 6hrs). Circles are experimental data. Black line: mechanistic model $T_c = 9.42 \cdot 10^{-5} A^{0.31}$ (SI). Dashed line: best-fitted line based on data $T_c = 4.71 \cdot 10^{-6} \cdot A^{0.56}$ (fitted as a power law, SI).

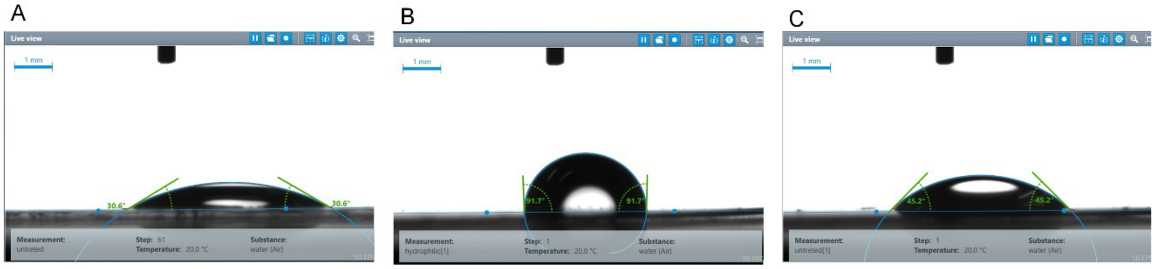

Fig. S7. Contact angle measurements on glass and modified surfaces. To mimic experimental conditions, 11µL drops of M9 medium supplemented with 20mM glucose were left for 3 hrs at 28°C, 100% RH. Contact angles were measured with a goniometer (EasyDrop DSA20E, KRÜSS GmbH, Hamburg, Germany). **A.** Untreated glass: 32.34° ± 2.55 (mean+SD, n = 6). **B.** Treatment 1 (Hydrophobic): 90.01° ±1.97 (n = 3) **C.** Treatment 2 (hydrophilic): 42.15° ± 4.2 (n = 4).

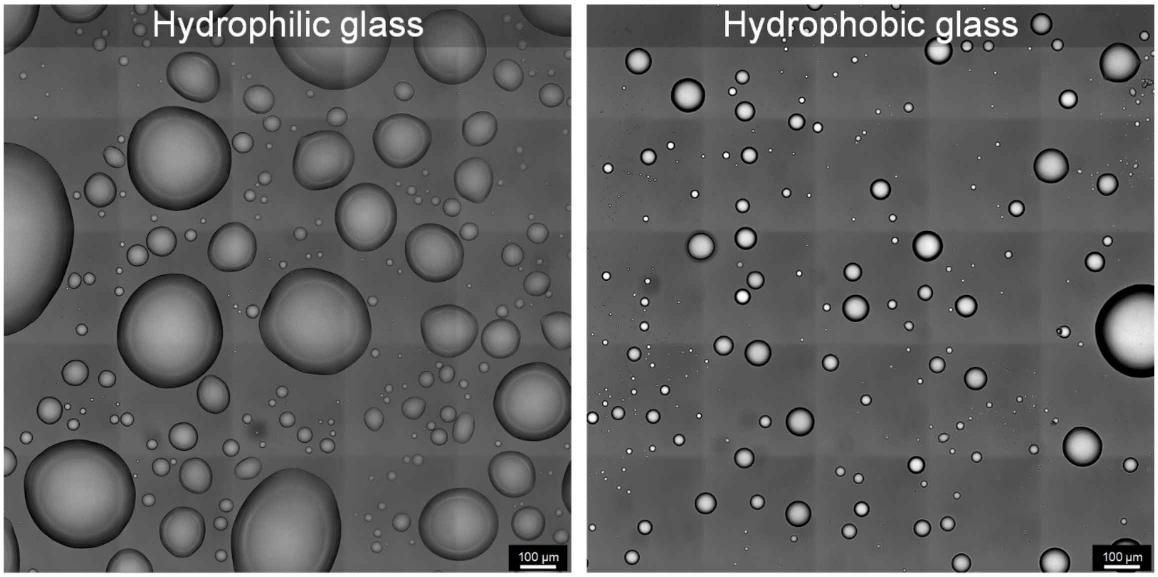

Fig. S8. Representative images of sprayed droplets deposited on (a) hydrophilic- and (b) hydrophobic-treated glass. Image constructed by stitching 5 × 5 adjacent fields of views into a single image (1.6 × 1.6 mm).

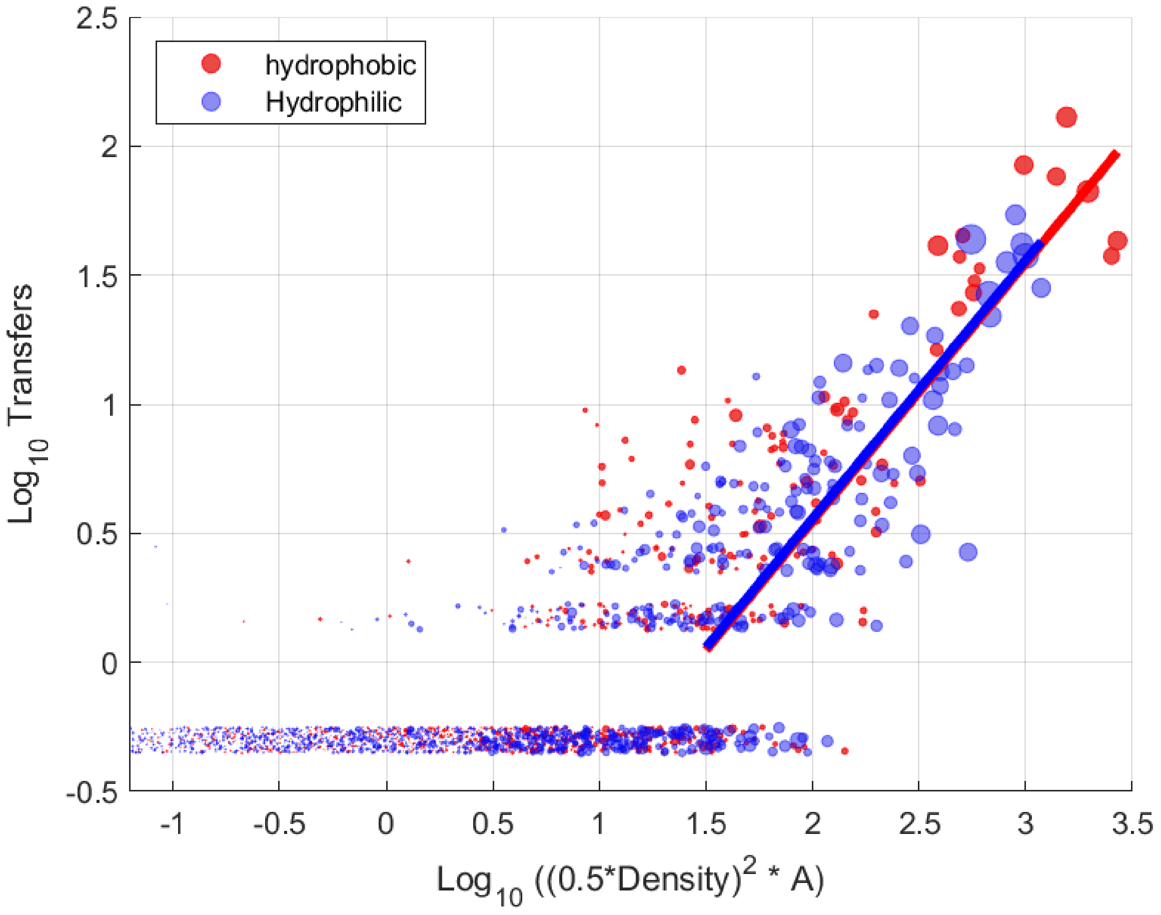

Fig S9. Mechanistic Density-based model of Transfer events in Experiment 2: treated surfaces (t = 6hrs). Axes are in log-log scales. Straight line depicts fitted model based on 'fitnlm' Matlab function to $Y = kX$. Red (hydrophobic) surface ($k1$ = 0.035±0.002); Blue (hydrophilic) surface ($k2$ = 0.036±0.001). Note that the fitted slope (k) is very similar for both treatments (is not statistically different, 2 sample t-test).

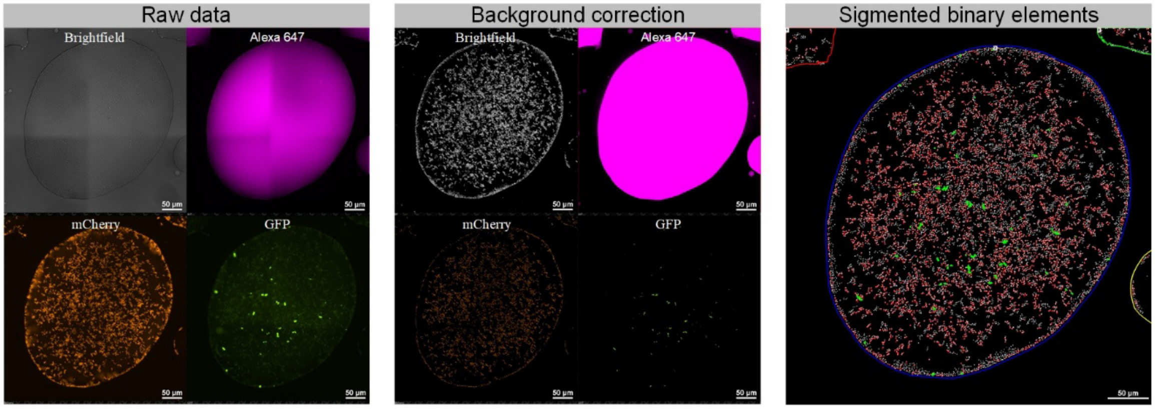

Fig. S10. Image processing steps. **Left:** Split-channel view of raw data image. Brightfield captures the droplet and the total cells within. Alexa 647 channel captures the droplet boundaries. mCherry channel captures the donor cells. GFP channel captures trans-conjugant cells. **Middle:** Rolling ball background correction was applied as a preprocessing step. **Right:** overlay of segmented elements from captured channels. Total bacterial cell in white, donor cell in red, and trans-conjugant cells in green.

Droplet ID	No. of cells at 3hrs (post inoculation)	No. of cells at 6hrs	No. of cells at 12hrs	No. of cells at 18hrs	Log10 drop area [µm²]	Mean no. of cell divisions (per cell)
1	5,554	6,590	7,836	7,951	4.80	0.72
2	226	313	358	362	3.76	0.80
3	321	467	607	609	3.97	0.95
4	15,214	17,555	21,430	20,702	5.09	0.68
5	2,930	3,746	4,963	4,789	4.62	0.81
6	878	1,239	1,507	1,473	4.27	0.84
7	1,833	2,465	3,134	3,040	4.53	0.83

Table S1. Number of cells in individual droplets over time, showing less than one cell division (on average) between 3 hrs and 18 hrs.

References

1. Klümper, U., Riber, L., Dechesne, A., Sannazzarro, A., Hansen, L.H., Sørensen, S.J., and Smets, B.F. (2015). Broad host range plasmids can invade an unexpectedly diverse fraction of a soil bacterial community. The ISME Journal *9*, 934.
2. Boks, N.P., Norde, W., van der Mei, H.C., and Busscher, H.J. (2008). Forces involved in bacterial adhesion to hydrophilic and hydrophobic surfaces. MICROBIOLOGY *154*, 3122-3133.
